# Turning Ultra‐Low Coercivity and Ultra‐High Temperature Stability Within 897 K via Continuous Crystal Ordering Fluctuations

**DOI:** 10.1002/advs.202402162

**Published:** 2024-05-06

**Authors:** Runqiu Lang, Haiyang Chen, Jinrong Zhang, Haipeng Li, Defeng Guo, Jianyuan Kou, Lei Zhao, Yikun Fang, Xiaoqiang Wang, Xiwei Qi, Yan‐dong Wang, Yang Ren, Haizhou Wang

**Affiliations:** ^1^ National Center for Materials Service Safety University of Science and Technology Beijing Beijing 100083 China; ^2^ Beijing Advanced Innovation Center for Materials Genome Engineering State Key Laboratory for Advanced Metals and Materials University of Science and Technology Beijing Beijing 100083 China; ^3^ Institute for Materials Intelligent Technology Liaoning Academy of Materials Shenyang 110004 China; ^4^ School of Materials Science and Engineering Northeastern University Shenyang 110819 China; ^5^ Functional Materials Research Institute Central Iron and Steel Research Institute Beijing 100081 China; ^6^ State Key Laboratory of Metastable Materials Science and Technology Yanshan University Qinhuangdao 066004 China; ^7^ College of Science Yanshan University Qinhuangdao 066004 China; ^8^ Beijing Advanced Innovation Center for Materials Genome Engineering Beijing Key Laboratory of Metal Materials Characterization Central Iron and Steel Research Institute Beijing 100081 China; ^9^ School of Resources and Materials Northeastern University at Qinhuangdao Qinhuangdao 066004 China; ^10^ Department of Physics City University of Hong Kong Hong Kong SAR 999077 China

**Keywords:** complex concentrated alloys, continuous crystal ordering, hierarchical microstructures, soft magnetic properties

## Abstract

High‐performance soft magnetic materials are important for energy conservation and emission reduction. One challenge is achieving a combination of reliable temperature stability, high resistivity, high Curie temperature, and high saturation magnetization in a single material, which often comes at the expense of intrinsic coercivity–a typical trade‐off in the family of soft magnetic materials with homogeneous microstructures. Herein, a nanostructured FeCoNiSiAl complex concentrated alloy is developed through a hierarchical structure strategy. This alloy exhibits superior soft magnetic properties up to 897 K, maintaining an ultra‐low intrinsic coercivity (13.6 A m^−1^ at 297 K) over a wide temperature range, a high resistivity (138.08 µΩ cm^−1^ at 297 K) and the saturation magnetization with only a 16.7% attenuation at 897 K. These unusual property combinations are attributed to the dual‐magnetic‐state nature with exchange softening due to continuous crystal ordering fluctuations at the atomic scale. By deliberately controlling the microstructure, the comprehensive performance of the alloy can be tuned and controlled. The research provides valuable guidance for the development of soft magnetic materials for high‐temperature applications and expands the potential applications of related functional materials in the field of sustainable energy.

## Graphical abstract

The strategy is to achieve comprehensive and superior magnetic properties through the nanostructured idea. The optimization of novel alloys through synergistic modulation of structure and chemistry at the atomic scale is leaving the shackles of the traditional view. The emergence of new ideas and strategies effectively supports the accelerated screening of high‐performance metallic materials.

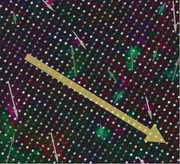

## 1 Introduction

Soft magnetic materials (SMMs) with a wider (or higher) service temperature range can lead to simpler, more efficient, and environmentally friendly designs that are important for sustainable energy and carbon neutrality.^[^ ^1^, ^2^, ^3^ ^]^ In application scenarios where potential high temperature environments exist, such as spacecraft power systems, ion propulsion engines, high energy density power supplies and energy storage devices, the key issues for soft magnetic properties can be specified as three points: 1) good temperature stability, which determines the service safety evaluation; 2) low intrinsic coercivity (_i_ *H*_c_) with high resistivity (*ρ*), which reduces core loss; and 3) sufficient saturation magnetization (*M*_s_), which improves energy storage density.^[^ ^4^, ^5^ ^]^ According to the Slater‐Pauling curve, Co‐doping is a common method to enhance temperature stability.^[^ ^6^, ^7^, ^8^ ^]^ Yet, for crystalline alloys such as Fe, Fe‐Si, Fe‐Al, etc., relying on Co‐doping alone leads to a significant degradation of _i_ *H*_c_ due to the increase in magnetostriction and magneto‐crystalline anisotropy.^[^ ^9^, ^10^ ^]^ Similarly, for amorphous/nanocrystalline (AN) alloys, Co‐containing HITPERM can extend the service temperature range to 797–897 K, which is a significant improvement over FINEMET and NANOPERM below 497 K.^[^ ^11^, ^12^, ^13^ ^]^ However, since Co‐doping leads to a harsh and uncontrollable nanocrystallization process within the amorphous matrix during fabrication, according to the Grain Size Dependence of Coercivity (GSDC) theory, this improvement comes at the cost of a relatively high *H*_c_ (≈26 A m^−1^).^[^ ^14^, ^15^, ^16^, ^17^, ^18^, ^19^ ^]^ Indeed, the hard magnetism Fe_2_B phase that easily precipitates in the metastable amorphous matrix during heating and the limitation of the first/second crystallization peak leave the application of AN alloys at elevated temperatures open to further discussion.^[^ ^15^, ^20^, ^21^, ^22^, ^23^ ^]^ In short, this scenario epitomizes the intricate challenge of regulating temperature stability, Curie temperature (*T*_c_) and *M*_s_ at the expense of high _i_ *H*_c_, a common trade‐off in the family of SMMs.^[^ ^15^ ^]^ Current constraints arise because conventional dilute alloys cannot perform multiple functions when tapping only from a limited compositional and phase space, which reduces the degrees of freedom for realizing both _i_ *H*_c_, temperature stability, *ρ* (which are more modulated by microstructural issues), *M*_s_ and *T*_c_ (which depend on intrinsic magnetism) required microstructures.^[^ ^9^, ^10^, ^24^, ^25^ ^]^ Therefore, it is worth considering the construction of appropriate microstructures or nanostructures in complex concentrated alloys (CCAs) with proper chemical composition to comprehensively break the shackles of coordination contradictions for the preferable performance of soft magnetic properties.

CCAs exhibit a wide variety of element occupations in the crystal lattice and possess numerous unusual physicochemical properties.^[^ ^26^, ^27^, ^28^ ^]^ These alloys have opened new avenues for the novel design of high‐performance SMMs through the synergistic regulation of micron‐ or nano‐scale crystal and chemical ordering structure.^[^ ^29^ ^]^ For example, by controlling the distribution of particles in the matrix, CCAs can achieve extremely low _i_ *H*_c_.^[^ ^30^, ^31^ ^]^ In addition, through continuous phase transition or spinodal decomposition, alloys can exhibit unconventional thermal‐stable magnetization.^[^ ^32^, ^33^ ^]^ Though constructing precipitates with a large aspect ratio form a Widmanstätten pattern in a strong and ductile multicomponent alloy, FeCoNiTa alloys achieve excellent high‐temperature magnetic and mechanical properties, not found in reference alloys of the same composition and without precipitates.^[^ ^34^ ^]^ Previously, we constructed an ordered dual‐phase nanostructure with an atomistic‐scale coherent interface in Ni_43_Fe_18_Ga_27_Co_12_ alloy to induce continuous lattice elastic distortion under applied loading.^[^ ^35^ ^]^ This was achieved by uniformly embedding the continuous ordered ω‐like phase in the *L*2_1_ superlattice, resulting in supercritical elastic behavior. Drawing inspiration from these advancements, we then propose four guidelines to expect superior soft magnetic properties (including but not limited to high‐temperature soft magnetic properties) for CCAs. First, achieving minimal domain wall pinning necessitates a well‐controlled configuration of internal interfaces in crystalline materials. Second, selecting an appropriate alloy composition is critical to maximize the magnetic moment. Third, reducing magneto‐crystalline anisotropy is essential to provide potential sites for reverse domain nucleation and propagation. Finally, incorporating a well‐tuned multi‐magnetic‐phase (state) nature is necessary to withstand magnetism degradation during the heating process. Intriguingly, the influence of constructing an appropriate atomistic‐scale entangled structure in ferromagnetic CCAs based on the crystal ordering/disordering modulation approach is not well comprehended, which hinders the discovery of potential synergistic optimization strategies.

It is well known that Fe‐Co alloys are the mainstay of soft magnetic materials for high‐temperature applications and that *B*2 ordering significantly enhances their temperature stability, *T*_c_ and *M*_s_, whereas in Fe‐Al alloys these properties are drastically reduced with *B*2 and *D*0_3_ ordering.^[^ ^4^, ^8^, ^36^ ^]^ Similarly, the Co_2_FeSi with *L*2_1_ (*cF*16‐AlCu_2_Mn type) exhibits the highest molecular magnetic moment (6 µB) among Heusler alloys when highly ordered, the highest *T*_c_ (1100±20 K), as well as favorable temperature stability within *T*_c_.^[^ ^37^, ^38^, ^39^ ^]^ The same is true for the regulation of the order degree in Co_2_FeAl.^[^ ^37^, ^38^, ^39^ ^]^ Based on these results and the GSDC theory, the entangled structure formed by highly ordered and partially ordered superlattices seems to be able to combine ultra‐high temperature stability, ultra‐low _i_ *H*_c,_ and moderate *M*_s_ in ferromagnetic materials. Moreover, the potential impact of atomic‐level order/disorder on the enhancement of *ρ* is intriguing. Inspired by these insights, we delve FeCoNiSiAl CCA as a model material. Since the dual‐phase nanostructure with continuous coherent interfaces in CCAs is inherently stable, while the long‐range ordering process requires a lengthy period of thermal preservation (above 1300 K), CCAs are expected to exhibit unique soft magnetic properties from room temperature (297 K) to high temperature (797–897 K).^[^ ^30^, ^31^, ^33^, ^34^, ^40^, ^41^ ^]^ In the following sections, we unveil an innovative design concept incorporating a hierarchical microstructure, which enables the construction of an atomic‐level order/disorder entangled structure through continuous crystal ordering (CCO) fluctuations. Such microstructural features realize a dual‐magnetic‐state nature with exchange softening, which can be predicted and controlled for _i_ *H*_c_ and temperature stability by deliberately manipulating the long‐range ordering process. Thus, this design strategy expands the application scenarios of crystal ordering/disordering modulation to optimize the functional properties of materials, and the new metallic material exemplifies a counterintuitive combination of ultra‐low _i_ *H*_c_, ultra‐high temperature stability, capable *ρ*, and moderate *M*_s_.

## 2 Results and Discussion

To elucidate the relationship between the process of long‐range ordering and the resulting microstructural variations, we selected five distinct states for comparative analysis. Figure S1a (Supporting Information) depicts the preparation route of five samples. Ingots with a nominal composition of Fe_49.45_Co_22.19_Ni_2.94_Si_16.09_Al_9.33_ (at. %) that underwent a stress‐relief treatment (detailed in the Experimental Section; the treatment for subsequent control samples remain consistent and will not be reiterated), are denoted as C‐CCA. M‐CCA samples were prepared from C‐CCA by melt spinning. In another preparation route, homogenized ingots were heat treated at 1523 K for 2 h followed by rapid quenching in water or prolonged annealing at 1523 K for 12 h with subsequent cooling in the furnace, yielding two varieties labeled Q‐CCA and A‐CCA, respectively. Further, Q‐CCA was ground into fine powder by vibratory milling; the fraction with particle sizes between 15 and 75 µm was then consolidated by spark plasma sintering (SPS), culminating in what is referred to as S‐CCA. Finally, homogenized ingots subjected to prolonged annealing at 1523 K for 500 h followed by slow cooling at a rate of 1 K per minute are classified as L‐CCA.

### 2.1 Continuous Crystal Ordering Fluctuations

**Figure 1:**
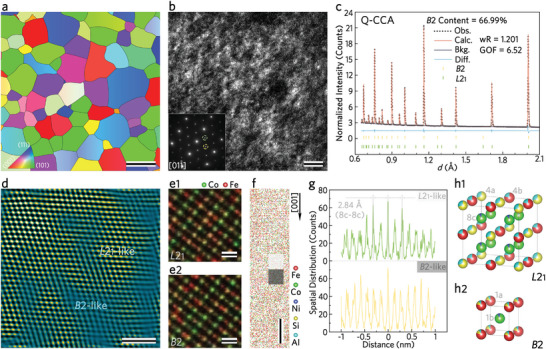
Hierarchical microstructure and CCO fluctuations of the Q‐CCA. a) IPF‐EBSD shows the coarse equiaxed grains. The thick black lines highlight the high angle grain boundaries. Scale bar, 1 mm. b) CDF‐TEM image of the *L*2_1_ nanodomains obtained by the (111) superlattice spot (see green circle in inset). The yellow and green circles indicate two different types of superlattice reflections: (200) for NN order and (111) for NNN order, respectively. Scale bar, 10 nm. c) Neutron scattering spectrum by Rietveld Refinement method for full pattern fitting, indicating that there are more CCO fluctuations than A‐CCA (as shown in Figure S2c, Supporting Information). d) IFFT image obtained from (111) showing the entangled structure of *B*2 and *L*2_1_ at the atomic scale. The unambiguous region corresponds to the *L*2_1_‐like zone, while the highly distorted region corresponds to the *B*2‐like zone. It is derived from the HAADF‐STEM image (Figure S2d, Supporting Information). Scale bar, 2 nm. e1), e2) Magnified image of the *L*2_1_‐like and *B*2‐like regions from the AR‐EDS map (Figure S2e, Supporting Information), showing that the *B*2‐like region is qualitatively Co‐rich. Scale bar, 4 Å. f) 3D reconstruction map (5 × 5 × 20 nm^3^) extracted from a typical tip by APT. The extracted area is indicated by a gray rectangle in Figure S2h (Supporting Information). The light and dark gray squares in this Figure indicate the *L*2_1_‐like and *B*2‐like regions analyzed in Figure 1g, respectively, with a size of 2 × 2 × 2 nm^3^. Scale bar, 3 nm. g) Spatial distribution curves of Co atoms taken along the [001] crystal orientation, showing the different regularity of Co spacing in *L*2_1_ and *B*2 (light and dark gray squares highlight *B*2 and *L*2_1_ in (f), respectively). Co atoms are preferentially spaced by 0.5a (a = 5.86 Å) in the *L*2_1_‐like region, but not in the *B*2‐like region. h1,h2) Schematic crystal structures of *L*2_1_ and *B*2, respectively.

The modulation of long‐range ordering processes profoundly influences the formation of atomic‐scale entangled structures. As shown in Figure S1b (Supporting Information), each of the five samples exhibits (111) and (200) superlattice diffraction peaks, evidencing the emergence of ordered phases. The inverse pole figure (IPF) obtained from electron backscatter diffraction (EBSD) data, shown in **Figure** **1**a, indicates that the grain size of Q‐CCA ranges from ≈1 to 2 mm, exhibiting the absence of crystallographic texture. S‐CCA, derived from powder particles, manifests a grain size that closely matches the dimensions of its constituent particles (shown in Figure S1c, Supporting Information). Notably, both A‐CCA and L‐CCA exhibit grains that are easily discernible to the naked eye, a feature attributed to annealing at 1523 K. Central beam dark‐field (CDF) transmission electron microscopy (TEM) analysis provides further insight, where exposed nanodomains become apparent against the darker background when the (111) superlattice spot is used for imaging, as shown in Figure 1b. In contrast, when the (200) superlattice spot is used, there is significant exposure at almost every position (as shown in Figure S2a, Supporting Information). The selected area electron diffraction pattern of Q‐CCA (inset of Figure 1b) identifies the (200) spot–indicating of nearest neighbor (NN) ordering–with a yellow circle, while the (111) spot–indicating of next‐nearest neighbor (NNN) ordering–is encircled in green. The crystal structures of NN and NNN ordering are continuous on larger scales but differ in the degree of ordering on smaller scales. The application of CDF‐TEM principles reveals the existence of an atomic‐scale entangled structure within Q‐CCA, stemming from CCO fluctuations.

The investigation expanded to include the control group, comparing C‐CCA with M‐CCA. Utilizing the (200) spot in the CDF‐TEM analysis revealed a discernible presence of NN ordered phases in both materials (Figure S1d2, Supporting Information versus Figure S1e2, Supporting Information). Conversely, when the imaging converted to the (111) spot, only sparse occurrences of NNN ordered phases were noticed (Figure S1d1,e1, Supporting Information). Moreover, the M‐CCA sample is distinguished by its tiny and numerous distributions of anti‐phase boundaries in contrast to C‐CCA. The dimensions of the anti‐phase domains are 200 versus 50 nm in C‐CCA and M‐CCA, respectively (Figure S1d2, Supporting Information versus Figure S1e2 inset, Supporting Information). In C‐CCA and M‐CCA, the appearance of anti‐phase boundaries indicates that the NN ordering is very fast. Regardless of whether the (111) or (200) spot was used, the CDF‐TEM images of S‐CCA (Figure S1f1,f2, Supporting Information) display a consistency with those of Q‐CCA. This suggests that the SPS process did not markedly change the microstructural characteristics inherent to Q‐CCA.

Considering the varying degrees of crystal order in the phases, BCC structures (*A*2) can transition to *B*2, *D*0_3_, or *L*2_1_ phases, as well as Quaternary Heusler phase (Strukturbericht Designations), which may exhibit overlapping diffraction indices.^[^ ^39^ ^]^ As depicted in Figure S2b (Supporting Information), the Z‐contrast at the 8c lattice site is markedly distinct from that at the 4a or 4b sites, which are further distinguished by the marked differences between the 4a and 4b sites themselves. This observation suggests that Q‐CCA is primarily a mixture of *L*2_1_ and *B*2 phases. Hence, we can assert that the atomic‐scale entangled structures emerging from CCO fluctuations are not simply equivalent to ordered phases precipitating within a disordered matrix. They represent a complex coalescence of superlattices with varied degrees of crystal order, articulating a more intricate arrangement than previously recognized.

To further investigate the effect of the level of CCO fluctuations over the heat preservation time, Rietveld Refinement of neutron scattering was used to analyze the evolution of *L*2_1_ and *B*2.^[^ ^42^ ^]^ As shown in Figure 1c, the relative content of *B*2 is higher in Q‐CCA and lower in A‐CCA (cf. Figure S2c, Supporting Information). More atoms are involved in the formation of *L*2_1_ with the prolongation of the time at 1523 K. This suggests that the level of CCO fluctuations in FeCoNiSiAl CCA can be intentionally controlled by rationally designing the heat preservation time, which is inevitably related to the properties of the new alloy.

In addition, the structural and chemical features of the CCO fluctuations were investigated. The high‐angle annular dark‐field (HAADF) image obtained by scanning transmission electron microscopy (STEM), together with the atomic resolution energy dispersive spectroscopy (AR‐EDS) map (Figure S2d,e, respectively, Supporting Information), reveals the chemical and structural origin of the CCO fluctuations. Disparities in the extinction rules between the *L*2_1_‐like and *B*2‐like regions were captured by Fast Fourier Transform (FFT) analysis of selected areas in Figure S2d1,d2, Supporting Information, noting in particular the absence of NNN reflections within the *B*2‐like zone. Moreover, the inverse Fast Fourier Transform (IFFT) reconstruction (shown in Figure 1d) distinctly exhibits the distribution of CCO fluctuations. Isolating the NNN reflections with a mask clearly delineates the *L*2_1_‐like areas and identifies areas of significant distortion associated with *B*2‐like zones. It indicates that atomic‐scale entangled structures are ≈1–4 nm in size. This is consistent with the morphology observed in the original HAADF images prior to FFT conversion (Figure S2d, Supporting Information) and graphically demonstrates the intricately interwoven structure of *L*2_1_ and *B*2 at the atomic scale.

### 2.2 Dual‐Magnetic‐State Nature

The magnified image of the *L*2_1_‐like and *B*2‐like zones extracted from Figure S2e (Supporting Information) shows that the *B*2‐like zone is qualitatively Co‐rich. The occupancy inclination of Fe and Co atoms is shown in Figure S2e (Supporting Information). The curves shown in Figure S2f (Supporting Information) quantify the Z‐contrast of rows 4a‐4b (i.e., row 1a in *B*2) along the [011] direction, as recorded along the white rectangle in Figure S2d (Supporting Information). These curves confirm the atomistic chemical discrepancy between the *L*2_1_‐like and *B*2‐like regions shown in Figure S2e (Supporting Information). The corresponding lattice sketches are shown in Figure 1h1,h2, also inserted in Figure S2g (Supporting Information), showing the crystallographic structures of *L*2_1_ and *B*2, where the green spheres represent the Co‐rich atoms occupying position 8c, and the yellow and red represent the Si‐ and Fe‐rich atoms occupying positions 4a and 4b. When *B*2 disorder occurs in *L*2_1_, positions 4a and 4b are mixed and collectively referred to as 1a (i.e., they lose the NNN feature), while 8c is referred to as 1b (as shown in Figure 1h2). The curves shown in Figure S2g (Supporting Information) plot the proportion of Co atoms within the 8c‐4a‐4b row (i.e., the 1a‐1b row in *B*2) along the [100] axis, as observed within the white rectangle outlined in Figure S2e (Supporting Information). The above results clearly indicate that the occurrence of atomic‐scale CCO fluctuations is caused by atomic‐scale chemical inhomogeneities.

3D reconstruction of atom probe tomography (APT) from a needle tip was used to interpret chemical inhomogeneity features in real crystal space. As shown in Figure S2h (Supporting Information), Q‐CCA does not exhibit typical elemental segregation at the submicron scale. However, according to the spatial distribution curves of Co atoms (Figure 1g) in the atomic image extracted from the needle tip (5 × 5 × 20 nm^3^, Figure 1f), Co‐Co atoms with a spacing of 0.5a (a = 5.683 Å) tend to be misaligned along the [100] zone axis (*L*2_1_‐like region, highlighted by the light gray square in Figure 1f). Correspondingly, the *B*2‐like region (possibly disordered *A*2) does not show this feature. When each packet contains 50 measured atoms, the frequency distribution curves of Fe and Co are shown in Figure S2i (Supporting Information), revealing the phenomenon of atomic‐scale chemical segregation. In particular, the Fe/Co content shows bimodal distributions, with the Co content peaking at ≈20% and 32%, respectively. In other words, the feature of atomic‐scale chemical inhomogeneity can be further summarized as the dual‐magnetic‐state nature.

Consequently, differential scanning calorimetry (DSC) of Q‐CCA and A‐CCA shows two lambda‐shaped protuberances belonging to the second‐order phase transition at 983 and 1250 K, respectively (Figure S3, Supporting Information). Based on the aforementioned Rietveld refinement and chemical analysis, the Co‐rich *B*2 has a *T*_c_ of ≈1250 K, while the Co‐poor *L*2_1_ has a *T*_c_ of ≈980 K. In summary, the atomic‐scale entangled structure fulfills the requirement of GSDC theory for reducing _i_ *H*_c_, while the dual‐magnetic‐state nature meets the requirement for modulating the crystal ordering degrees to enhance temperature stability.

### 2.3 Soft Magnetic Responses

**Figure 2:**
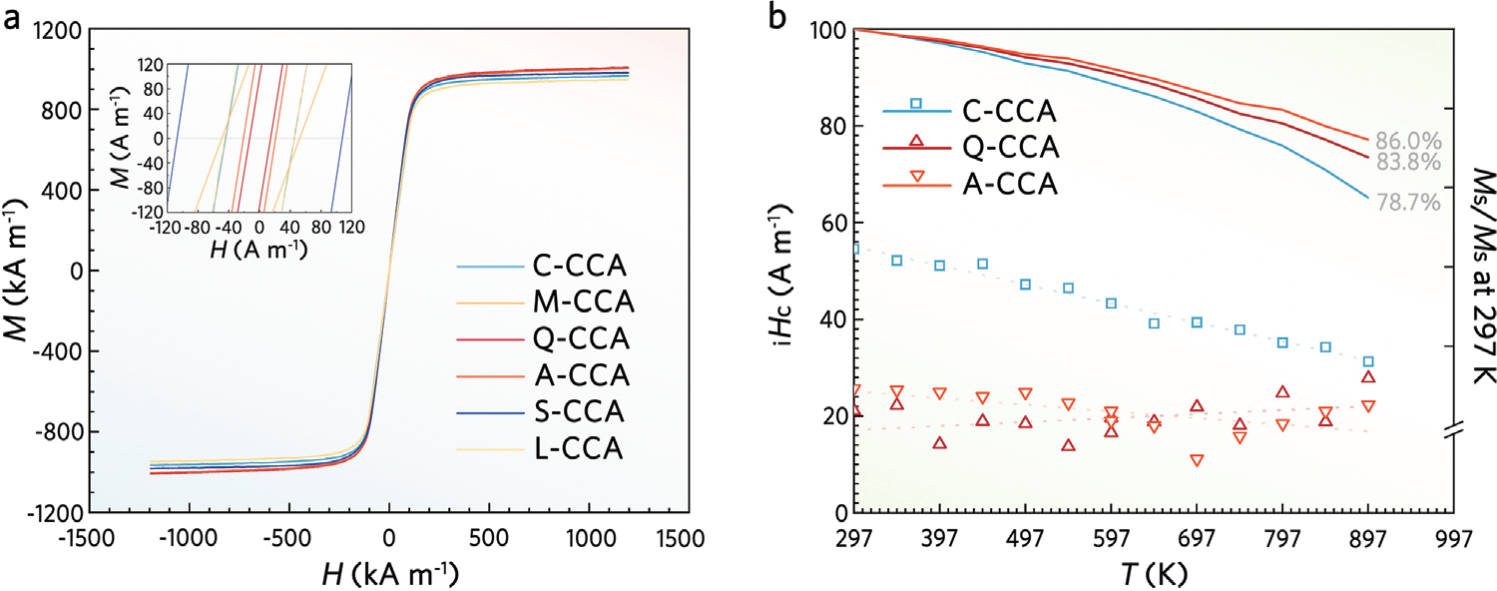
Soft magnetic responses of FeCoNiSiAl CCAs. a) Hysteresis loops (*M*‐*H*) recorded up to ±1200 kA m^−1^. The field sweep rate is 0.796 A m^−1^. The upper left inset shows the ultra‐low _i_ *H*_c_ of Q‐CCA. b) Temperature stability of C‐CCA, Q‐CCA, and A‐CCA. Data measured from in‐situ heating *M*–*H* curves to 897 with 50 K intervals. The left and right axes show the _i_ *H*_c_–*T* and *M*_s_–*T* curves, respectively. The Q‐CCA and A‐CCA have consistently shown thermally stable magnetization, with ultra‐low _i_ *H*_c_ and sufficient *M*_s_ within 897 K.

The *M*‐*H* hysteresis loop measured from five samples is shown in **Figure** **2**a. At 297 K, Q‐CCA and A‐CCA exhibited ultra‐low _i_ *H*_c_ of 13.6 and 20.9 A m^−1^, respectively. In comparison, the _i_ *H*_c_ of C‐CCA and L‐CCA were large, while the _i_ *H*_c_ of S‐CCA was the largest (Figure 2a, inset). This reflects the change in _i_ *H*_c_ controlled by microstructural factors with the long‐range ordering process of FeCoNiSiAl CCA. The density of the sample was measured by the buoyancy method to be ≈7058 kg m^−3^. The *M*_s_ were 1006.89 and 1003.93 kA m^−1^ for Q‐CCA and A‐CCA, respectively, and were essentially the same for the control samples. The small differences in *M*_s_ are due to the consistency of the chemical component, while sufficient *M*_s_ is due to sufficient ferromagnetic elements in the alloy composition, that is, Fe/Co/Ni content. The ultra‐low _i_ *H*_c_ of Q‐CCA supports the positive significance of regulating the level of CCO fluctuations. Although the existence of CCO fluctuations is a common feature of both Q‐CCA and A‐CCA, it is clear from the GSDC theory that accurate control of microstructural issues is essential.

To further verify the temperature stability of the soft magnetic properties at elevated temperatures, the in‐situ heating hysteresis loop (297 to 897 K, 50 K interval) of C‐CCA, Q‐CCA, and A‐CCA was measured. As shown in Figure 2b, the *M*_s_ of Q‐CCA is 839.20 kA m^−1^ at 897 K with an attenuation of only 16.7%, while C‐CCA and A‐CCA are 21.3% and 14.0%, respectively. A‐CCA and Q‐CCA show ultra‐high temperature stability, which also supports the positive significance of regulating the level of CCO fluctuations. During in‐situ heating, the _i_ *H*_c_ variations in Q‐CCA and A‐CCA are different from those in C‐CCA, which always remain in the lower range. The _i_ *H*_c_ in C‐CCA becomes gradually smaller with increasing temperature, which is in line with the usual phenomena of _i_ *H*_c_‐T relationship in magnetic materials, and the reason for this lies in the relationship between magneto‐crystalline anisotropy and magnetostriction with temperature. However, the temperature independent _i_ *H*_c_ manifestation that occurs in Q‐CCA and A‐CCA is due to the differences in their magnetization mechanisms, which will be discussed below.

### 2.4 Magnetic Domains and Dynamic Evolution

The question arises as to the origin of the remarkable properties of ultra‐high temperature stability and ultra‐low _i_ *H*_c_ observed in Q‐CCA and A‐CCA. To shed light on this question, we delve into the magnetization mechanism through the following investigation.

**Figure 3:**
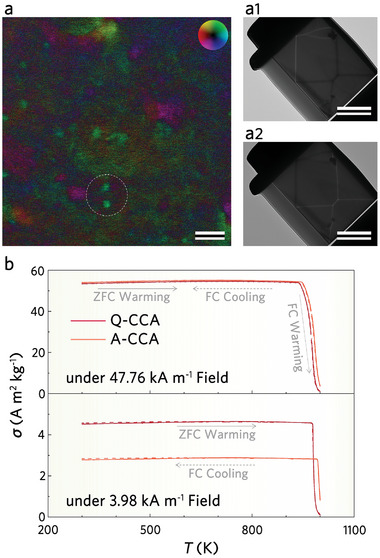
Observation of magnetic domains and dynamic evolution in Q‐CCA and A‐CCA. a) DPC‐STEM image of Q‐CCA showing the morphology of the entangled structure formed by CCO fluctuations at the atomic scale. The dispersion feature is highlighted by the white circle, indicating the local contrast otherness. (The color wheel in the legend represents the magnetic field direction). Scale bar, 10 nm. a1) L‐TEM under‐focus image. Scale bar, 2 µm. a2) L‐TEM over‐focus image. Scale bar, 2 µm. By combining the light and dark lines observed in (a1) and (a2), the domain walls can be observed under zero‐field conditions. b) ZFC and FC curves of Q‐CCA and A‐CCA, showing the difference in response between Q‐CCA and A‐CCA under a weak field (3.98 kA m^−1^).

Differential phase contrast (DPC) STEM was used to characterize the microscopic magnetic field distribution to gain more insight into the relationship between soft magnetic properties and microstructural issues. As shown in **Figure** **3**a, the white circles indicate that the scale of the local tinctorial undulations corresponds to the size of the CCO fluctuations, with the color wheel representing the field direction. Observed in Lorentz TEM (L‐TEM), the under‐focus (Figure 3a1) and over‐focus (Figure 3a2) images reveal the magnetic domain structure of the sample under zero field. When the electromagnetic field distribution inside crystalline samples is observed by DPC‐STEM, the DPC image intensity contains not only the contrast induced by the electromagnetic field but also the contrast induced by the diffraction condition.^[^ ^43^ ^]^ In Q‐CCA, where the electron beam is incident from the [011] zone axis, the contrast difference in the DPC image results from the overlap of the magnetic domains with the higher‐order Laue zone lines induced by the CCO fluctuations. It shows that the nanostructural units are ≈1–4 nm in size, consistent with that observed in HAADF‐STEM (Figure 1d). In fact, this provides a more intuitive representation of the atomic‐scale entangled structure, that is, the dual‐magnetic‐state nature, constructed via CCO fluctuations.

By applying the zero‐field cooling and field cooling (ZFC/FC) magnetization processes under the weak (3.98 kA m^−1^) and medium (47.76 kA m^−1^) fields to Q‐CCA and A‐CCA, it is found that the curves of the two samples basically coincide without divergence (Figure 3b). Under a weak field of only 3.98 kA m^−1^, the magnetization per unit mass (*σ*) values of Q‐CCA and A‐CCA are 4.68 and 2.89 A m^2^ kg^−1^, respectively. Apparently, the response of Q‐CCA to weak fields is more pronounced. Figure S4a (Supporting Information) shows the d*M*/d*T*–*T* curves of Q‐CCA versus A‐CCA under the field of 47.76 kA m^−1^, and the *T*_c_ of both appears at ≈980 K, which is consistent with the *T*_c_ of *L*2_1_ identified by DSC. As shown in Figure S4b (Supporting Information), the hysteresis loops of Q‐CCA and A‐CCA at 1000 K indicate that the ferromagnetism currently originates from *B*2. It is worth noting that the maximum value of *σ* in the *σ*–*H* curve is the magnetic moment under the field of 1200 kA m^−1^ divided by the mass of the whole sample, not the mass of *B*2 therein. In conclusion, A‐CCA exhibits better temperature stability than Q‐CCA due to the fact that the prolonged long‐range ordering process allows more Si and Al to participate in the formation of *L*2_1_, resulting in higher purity and ordering of *B*2.

Figure S5a (Supporting Information) shows the in‐situ domain evolution of non‐magnetized historical samples as observed by magneto‐optical Kerr effect microscopy. Upon application of a field ranging from 0 to 45%*M*_s_, the magnetic domains undergo progressive “dissolution” rather than the typical domain wall movement. At 63%*M*_s_, discernible domain walls are essentially absent, indicating that the magnetization directions of the individual domains are approximately the same. A distinctive striped domain morphology appears abruptly at 73%*M*_s_, with contrast stripes oriented parallel to the field direction. The image contrast stabilizes at 85%*M*_s_, indicating that magnetization is approaching saturation and that the domain is undergoing the final spin rotation. According to the minimum energy principle, when a field is applied to a domain, the magnetic moment of the domains will eventually change along the field direction.^[^ ^44^ ^]^ This process can occur through several mechanisms, that is, reversible domain wall movements, irreversible domain wall movements, and spin rotation.^[^ ^45^ ^]^ The in‐situ domain evolution in C‐CCA, as shown in Figure S5b (Supporting Information), follows the common mechanism of domain wall movement.^[^ ^46^ ^]^ Herein, with the application of the field, the domain walls gradually expand from one side to the other, roughly along the direction of the field. The opposing magnetic moment near the domain wall rotates toward the direction of the field, and the magnetic moment in the domain wall near the direction of the field rotates further, gradually leaving the transition layer of the domain wall into another magnetic domain. In contrast, Q‐CCA exhibits a domain motion mechanism dominated by domain spin rotation. In the first half of the magnetization, the domain walls barely move in response to the field. When a critical strength is reached (63%*M*_s_), the domain walls disappear instantaneously, leading to the merging of adjacent domains. A plausible explanation is that the atomic‐scale entangled structure achieves exchange softening and the smaller magneto‐crystalline anisotropy favors spin rotation. We emphasize that this spin rotation dominated mechanism is rarely observed in previous studies of SMMs.^[^ ^47^ ^]^

### 2.5 Exchange Softening

We believe that the hierarchical structure and dual‐magnetic‐state nature play a crucial role in maintaining the ultra‐low _i_ *H*_c_ and ultra‐high temperature stability within 897 K. GSDC theory effectively explains the magnetization mechanism in AN alloys.^[^ ^16^, ^17^, ^18^, ^19^ ^]^ When the structural correlation length (*D*) is considerably smaller than the basic exchange length (*L*_0_), the orientation of magnetic moments within a single domain depends on the competition between the exchange energy of each structural unit and its own magnetic anisotropy energy, thus preventing the magnetization from following the easy axis of each individual structural unit.^[^ ^16^, ^17^ ^]^ It is clear that the atomic‐scale entangled structure of Fe‐based alloys, with an *L*_0_ of ≈40 nm, facilitates the achievement of penetrating exchange with *D* < *L*_0_. In addition, CCO fluctuations arise from atomistic level chemical inhomogeneities. Despite their continuous crystal structures, numerous *L*2_1_, *B*2, and a trace amount of *A*2 (i.e., structural units) within a single grain do not follow the same easy axis of magnetization as an ideal crystal. This mechanism leads to a reduction in the net magnetic anisotropy, which is averaged over multiple structural units and consequently reduced in magnitude. In short, the entangled structure achieves exchange softening, resulting in the dominance of ferromagnetic exchange interactions. As a result, the negative effect of Co‐doping is mitigated, that is, its positive effect of improving temperature stability and increasing *M*_s_ is simultaneously obtained, while maintaining a stable ultra‐low _i_ *H*_c_ that is almost independent of temperature change.

It is important to emphasize that the suppression of random magneto‐crystalline anisotropy by exchange interaction is effective over a wide temperature range, which is the key factor that distinguishes Q‐CCA and A‐CCA from C‐CCA.^[^ ^17^, ^48^, ^49^ ^]^ The ability of FeCoNiSiAl CCA to maintain ultra‐low _i_ *H*_c_ and ultra‐high temperature stability within 897 K is mainly due to the fact that the exchange softening mechanism is not limited by the magneto‐crystalline anisotropy of single‐phase magnetic crystals with respect to temperature.^[^ ^17^ ^]^ This fundamental difference distinguishes the application of hierarchical structural strategies in CCAs from doping methods that seek the composition with the minimum magneto‐crystalline anisotropy and magnetostriction in conventional dilute alloys. In the alloy studied above, as the temperature approaches the first *T*_c_ (≈973 K), the inter‐superlattice coupling between the entangled structures is significantly weakened due to the rapid decay of the ferromagnetism in *L*2_1_, leading to a correspondingly rapid degradation of the soft magnetic properties. At 1000 K, the presence of residual ferromagnetism indicates that the exchange penetration achieved by the entangled structure has not been completely interrupted. In fact, as the temperature increases, both the coupling strength and the *M*_s_ of *B*2 decrease simultaneously, leaving the precise coupling mechanism an intriguing mystery.

### 2.6 Nature of Temperature Stability

According to Weiss theory, the magnetization of a single phase ferromagnet gradually decreases with temperature, approaching *T*_c_ where it deteriorates significantly, which can be well fitted by the Brillouin function.^[^ ^9^, ^50^ ^]^ The relationship between the valence electron number and the magnetic moment of transition metals can be described by the well‐established Slater‐Pauling curve.^[^ ^6^, ^7^ ^]^ In short, the utilization of the Fe‐Co coupling effect is necessary to obtain reliable temperature stability with a sufficient amount of *M*_s_. Unlike the Co‐doping of dilute alloy systems (Fe‐based, Fe‐Si, Fe‐Si‐Al), the role of the Co element in CCA is more to give the alloy system the freedom to form NN‐order versus NNN‐order phases. In Q‐CCA and A‐CCA, the change in the relative content of *L*2_1_ and *B*2 is directly reflected in the temperature stability. For C‐CCA, the long‐range ordering process is moreover able to directly highlight the positive significance (Figure 2b). The Co‐rich *B*2 compensates to some extent for the rapid demagnetization of the Co‐poor *L*2_1_ caused by the temperature rise. *L*2_1_ and *B*2, both with reliable temperature stability, precede and follow each other over a wide temperature range, resulting in ultra‐high temperature stability within 897 K.

### 2.7 Long‐Range Ordering Process

Structure determines performance, and magnetic domain structure determines soft magnetic properties. The _i_ *H*_c_ is the overall measure of various irreversible processes during the magnetization of magnetic materials.^[^ ^50^, ^51^ ^]^ In real materials, sites with low magneto‐crystalline anisotropy can serve as potential nucleation and propagation points for reverse domains or act as barriers to domain wall motion.^[^ ^51^, ^52^, ^53^ ^]^ Based on the above discussion, the _i_ *H*_c_ and temperature stability of FeCoNiSiAl CCA can be determined by controlling the long‐range ordering process. For example, a comparison between S‐CCA and Q‐CCA shows that the abundant presence of grain boundaries directly causes the pinning effect of domain wall movement. In other words, S‐CCA is only regulated at the nanoscale, without hierarchical structural control. When Q‐CCA, A‐CCA, and L‐CCA are compared laterally, although all three have achieved a hierarchical structure by constructing millimeter‐scale non‐oriented equiaxed crystals, the exchange softening effect is weakened, leading to the degradation of _i_ *H*_c_ due to excessive ordering. In addition, the room‐temperature *ρ* of C‐CCA, Q‐CCA, S‐CCA, and A‐CCA were measured by the four‐probe method, and the values are 114.58, 138.08, 116.97 and 88.60 µΩ cm, respectively. Taking SuperSendust (Fe‐8Si‐4Al‐3.2Ni, *ρ* = 100 µΩ cm) with its high *ρ* in the family of soft magnetic alloys as a reference, although the Si and Al contents in A‐CCA are higher than those in SuperSendust, the long‐range ordering process results in slightly lower *ρ*.^[^ ^54^ ^]^ This further proves that in CCAs designed based on a hierarchical structural strategy, proper control of the microstructure is still crucial, despite the ability to exploit the freedom afforded by multiscale crystal and chemical ordering structures.

### 2.8 Soft Magnetic Properties

**Figure 4:**
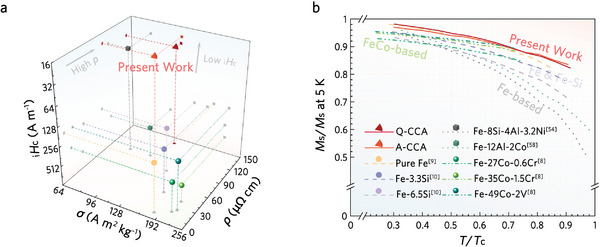
Soft magnetic properties and temperature stability combined in the novel Q‐CCA and A‐CCA. a) Ashby map plotting *M*_s_ versus _i_ *H*_c_ at 897 K and *ρ* at 297 K compared to the typical soft magnetic alloys, such as pure Fe, Fe‐Si, Fe‐Al‐Co, Fe‐Co‐X, and SuperSendust, which can still serve at 897 K. Legends are shown below. b) Relative magnetization *M*_s_/*M*_s_ at 5 K as a function of relative temperature *T*/*T*_c_ compared to those of the other typical soft magnetic alloys. The *T*_c_ of Q‐CCA and A‐CCA were chosen to be the *T*_c_ of their respective *L*2_1_.

To highlight the fine combination of *M*_s_, _i_ *H*_c_ versus *ρ* in Q‐CCA and A‐CCA, we compare them to typical SMMs that can serve at 897 K (**Figure** **4**a). The _i_ *H*_c_ and *M*_s_ in this figure are the soft magnetic properties of these alloys at 897 K, and *ρ* is the room temperature data. 897 K is slightly higher than the highest temperature at which AN soft magnetic alloys do not precipitate Fe_2_B hard ferromagnet.^[^ ^55^, ^56^, ^57^ ^]^ Figure 4b illustrates the *M*_s_/*M*_s_ at 5 K‐*T*/*T*_c_ curve for the alloys in Figure 4a. The *M*_s_ and _i_ *H*_c_ values of these soft magnetic alloys being compared at 897 K were obtained from commercial materials using the same test method with the same sample preparation conditions, and the room‐temperature *ρ* was taken from Ref. [58]. Meanwhile, the room temperature *M*_s_ (or saturation flux density) and *H*_c_ with coercivity (*H*_c_) or _i_ *H*_c_ mentioned in Ref. [4, 5, 8, 9, 10, 17, 18, 50, 51, 54, 58] were used to verify the quality of the commercial materials. Compared to most existing soft magnetic alloys, the current alloys (marked with red tetrahedrons) exhibit ultra‐low _i_ *H*_c_ and ultra‐high temperature stability within 897 K. In addition, the *M*_s_/*M*_s_ at 5 K‐*T*/*T*_c_ curves of Q‐CCA and A‐CCA demonstrate that the thermally stable magnetization of the current alloy is comparable to that of pure Fe (Figure 4b). Although Q‐CCA and A‐CCA are slightly inferior to conventional Fe‐Co alloys, they are significantly stronger than SuperSendust. Notably, as shown in Figure S6 (Supporting Information), the much lower _i_ *H*_c_ observed in the current alloy has not been observed in any other high‐entropy alloy reported to date (Supporting References for details).

## 3 Conclusion

In summary, we have discovered ferromagnets in complex concentrated alloys that combine ultra‐low intrinsic coercivity and ultra‐high temperature stability over a wide temperature range. We have successfully developed a new type of soft magnetic alloy, designed through a crystal order/disorder methodology, which features an atomic‐scale entangled structure induced by continuous crystal ordering fluctuations, thus achieving a dual‐magnetic‐state nature with exchange softening. Furthermore, by combining large‐scale non‐oriented equiaxed crystals, we have succeeded in constructing a hierarchical structure. By deliberately manipulating the long‐range ordering process of the alloy and exploiting the freedom afforded by the synergistic regulation of crystal and chemical ordering structures, we have achieved a counterintuitive combination of ultra‐low intrinsic coercivity, ultra‐high temperature stability, high resistivity, and moderate saturation magnetization–properties that are difficult to achieve simultaneously in conventional dilute alloy systems. The discovery of soft magnetic properties associated with continuous crystal ordering fluctuations paves the way for controlling certain properties and developing related functional materials through the entangled structure of order and disorder at the atomic scale in materials.

Complex concentrated alloys have unlimited compositional or structural space at the multiscale level, allowing the ingenious combination of many unusual physical and chemical properties. New alloy design methods can be tailored to the needs of the service environment to customize soft magnetic materials. Whether in the manufacturing process or in use, traditional soft magnetic materials always have some limitations in certain aspects. Future development of advanced high‐performance magnetic complex concentrated alloys could be based on the materials genome strategy, integrating high‐throughput fabrication and characterization, computational materials science, machine learning, and other frontier methods. This approach could accelerate the discovery of new alloy variants, and further improve soft magnetic properties (such as higher saturation magnetization strength), while reducing alloy costs.

## 4 Experimental Section

### Sample Preparation

The present alloys were synthesized by arc melting of high purity raw materials and then the ingots were placed in a Pyrex glass tube in a pure argon atmosphere. After homogenization heat treatment (1573 K/12 h), the ingot was water quenched together with the glass tube. The homogenized ingots were machined to the appropriate size by spark erosion, and the polished samples were etched with argon ions to remove the stress layer. The subsequent Q‐CCA, A‐CCA, and L‐CCA samples were again placed in a smaller Pyrex glass tube for final heat treatment. The inductively coupled plasma optical emission spectrometer was used to analyze the chemical composition of the samples.

### SEM & TEM Characterization

Electron backscatter diffraction images were obtained in the ZEISS SUPRA 55 scanning electron microscope at a voltage of 20 kV. The specimens of transmission electron microscopy were extracted from the de‐stressed bulk sample by a focused ion beam. To avoid the effect of Ga ion implantation, the final grinding step was performed with an ion beam at a current of 23 pA and an acceleration voltage of 2 kV. Atomic‐scale high‐angle annular dark‐field, energy dispersive spectroscopy & differential phase contrast, EDS & DPC images were obtained using an advanced double spherical aberration corrected scanning transmission electron microscope Thermo Scientific Spectra 300 (S)TEM.

### Neutron Scattering & XRD

The neutron scattering specimens were prepared by suction casting the homogenized ingots into 6 mm diameter rods. These rods were encapsulated in Pyrex tubes after de‐stressed treatment, using the same heat treatment process as mentioned above to maintain the same microstructure as the earlier small samples. Neutron scattering measurements were performed on the Multiphysics Spectrometer (MPI) at the China Spallation Neutron Source (CSNS). X‐ray diffraction (XRD) characterization of the phase was performed using an advanced high‐power X‐ray in‐situ characterization system (Rigaku Smartlab) with a Cu target.

### 3D APT Reconstruction & DSC

Tips for atom probe tomography analysis were prepared by focused ion beam milling using a Thermo Scientific Helios 5 DualBeam. The tips were characterized on the CAMECA LEAP 5000 XR in the electric field evaporation mode at 50 K, with a pulse rate of 200 kHz, a pulse fraction of 15%, and a detection rate of 0.005 atoms per pulse. The collected data were reconstructed and analyzed using CAMECA IVAS v.3.6.14 software. Simultaneous thermal analysis characterization was performed using SETARAM Labsys Evolution at a rate of 1 K min^−1^ and a test range of 297–1697 K.

### Soft Magnetic Response

The soft magnetic response was evaluated using the Quantum Design Physical Property Measurement System (PPMS) and the Lake Shore 8600 vibrating sample magnetometer (VSM), respectively. 3 × 2 × 1 mm^3^ (length × width × thickness) cubic de‐stressed specimens were used for the measurements. For the 2 × 3 × 1 mm^3^ sample, the 2 × 3 mm^2^ surface was fixed to the sample chamber, and the field was loaded perpendicular to the 3 × 1 mm^2^ surface. The soft magnetic response at high temperatures was tested using the Lake Shore 8600 86‐OVEN high‐temperature oven with GlideLOCK alignment system and the PPMS‐VSM oven. 5 × 3 × 1 mm^3^ (length × width × thickness) samples were used for the magneto‐optical Kerr effect observations using the same preparation procedure as above and the instrumentation used was evico magnetics GmbH/em‐Kerr‐highres.

## Conflict of Interest

The authors declare no conflict of interest.

## Supporting information

Supporting Information

## Data Availability

The data that support the findings of this study are available from the corresponding author upon reasonable request.
