# Supplementary material for: Turning Ultra‐Low Coercivity and Ultra‐High Temperature Stability Within 897 K via Continuous Crystal Ordering Fluctuations
Source: Adv Sci (Weinh). 2024 May 6;11(28):2402162. doi: 10.1002/advs.202402162 (PMC11267332; doi:10.1002/advs.202402162)
Supplement: Supplementary file 1 — Supporting Information [file ADVS-11-2402162-s001.pdf]

ADVANCED SCIENCE
Open Access

Supporting Information

for *Adv. Sci.*, DOI 10.1002/advs.202402162

Turning Ultra-Low Coercivity and Ultra-High Temperature Stability Within 897 K via Continuous Crystal Ordering Fluctuations

Runqiu Lang, Haiyang Chen, Jinrong Zhang, Haipeng Li, Defeng Guo, Jianyuan Kou, Lei Zhao, Yikun Fang, Xiaoqiang Wang, Xiwei Qi, Yan-dong Wang, Yang Ren and Haizhou Wang**

Supporting Information

Turning Ultra-Low Coercivity and Ultra-High Temperature Stability within 897 K via Continuous Crystal Ordering Fluctuations

*Runqiu Lang, Haiyang Chen, Jinrong Zhang, Haipeng Li, Defeng Guo, Jianyuan Kou, Lei Zhao, Yikun Fang, Xiaoqiang Wang, Xiwei Qi, Yan-dong Wang**, *Yang Ren, Haizhou Wang**

This file includes:

- Supporting Figures S1~S6
- Supporting References S1~S23

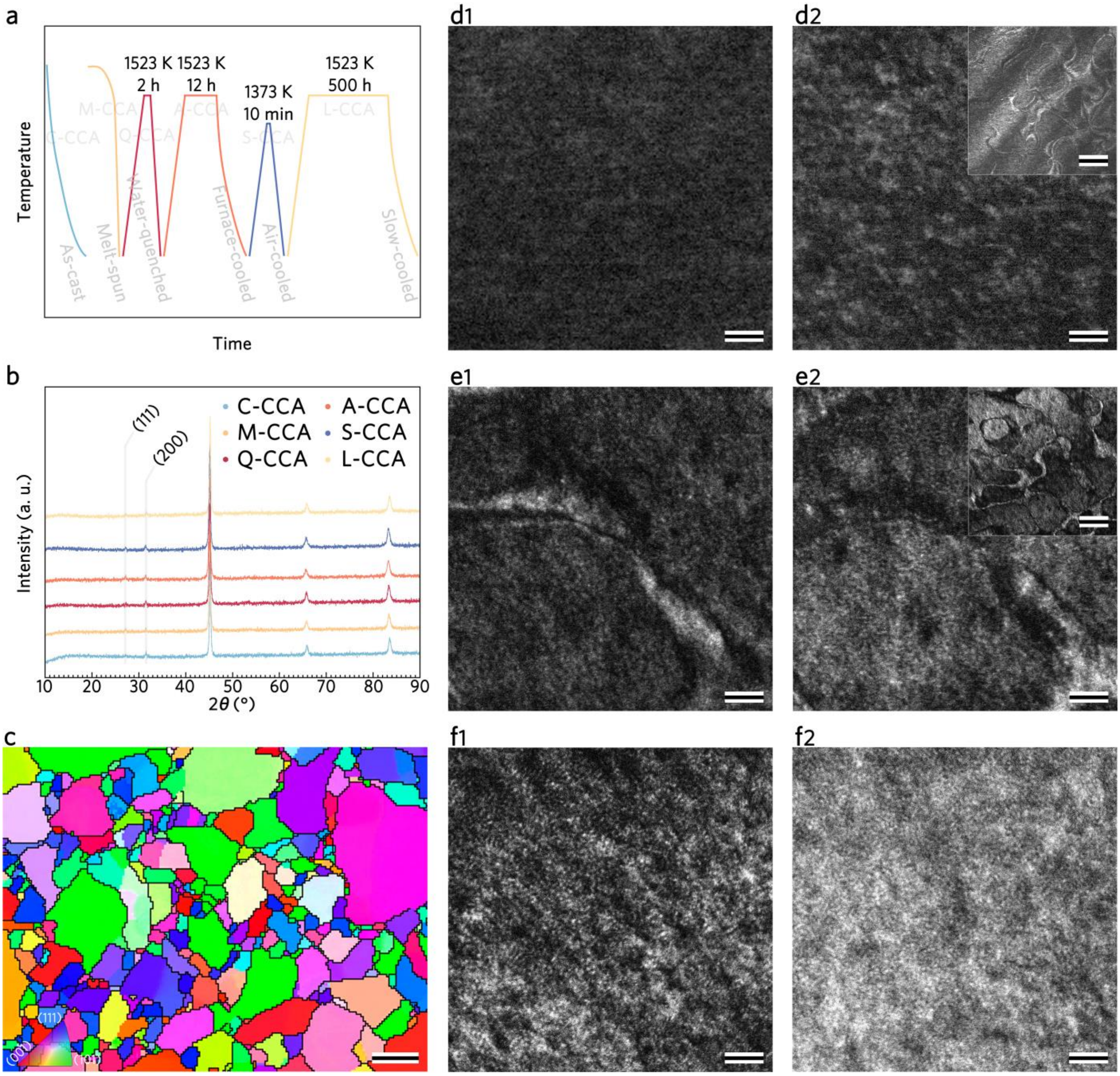

Figure S1. Microstructure of the control group. a) Schematic representation of the heat treatment routes for C-CCA, M-CCA, Q-CCA, A-CCA, S-CCA and L-CCA. b) XRD spectra of the control group. The presence of (111) and (200) was observed in all the samples. c) IPF-EBSD of S-CCA. The grain size is approximately the same as the powder particle size before sintering. Scale bar, 20 µm. d1), e1) & f1) CDF-TEM images obtained by the (111) superlattice spot in C-CCA, M-CCA and S-CCA, respectively. Scale bar, 10 nm. d2), e2) & f2) CDF-TEM images obtained by the (200) superlattice spot in C-CCA, M-CCA and S-CCA, respectively. Scale bar, 10 nm. Scale bar of the insets in d2) and e2) is 200 nm vs. 50 nm, respectively.

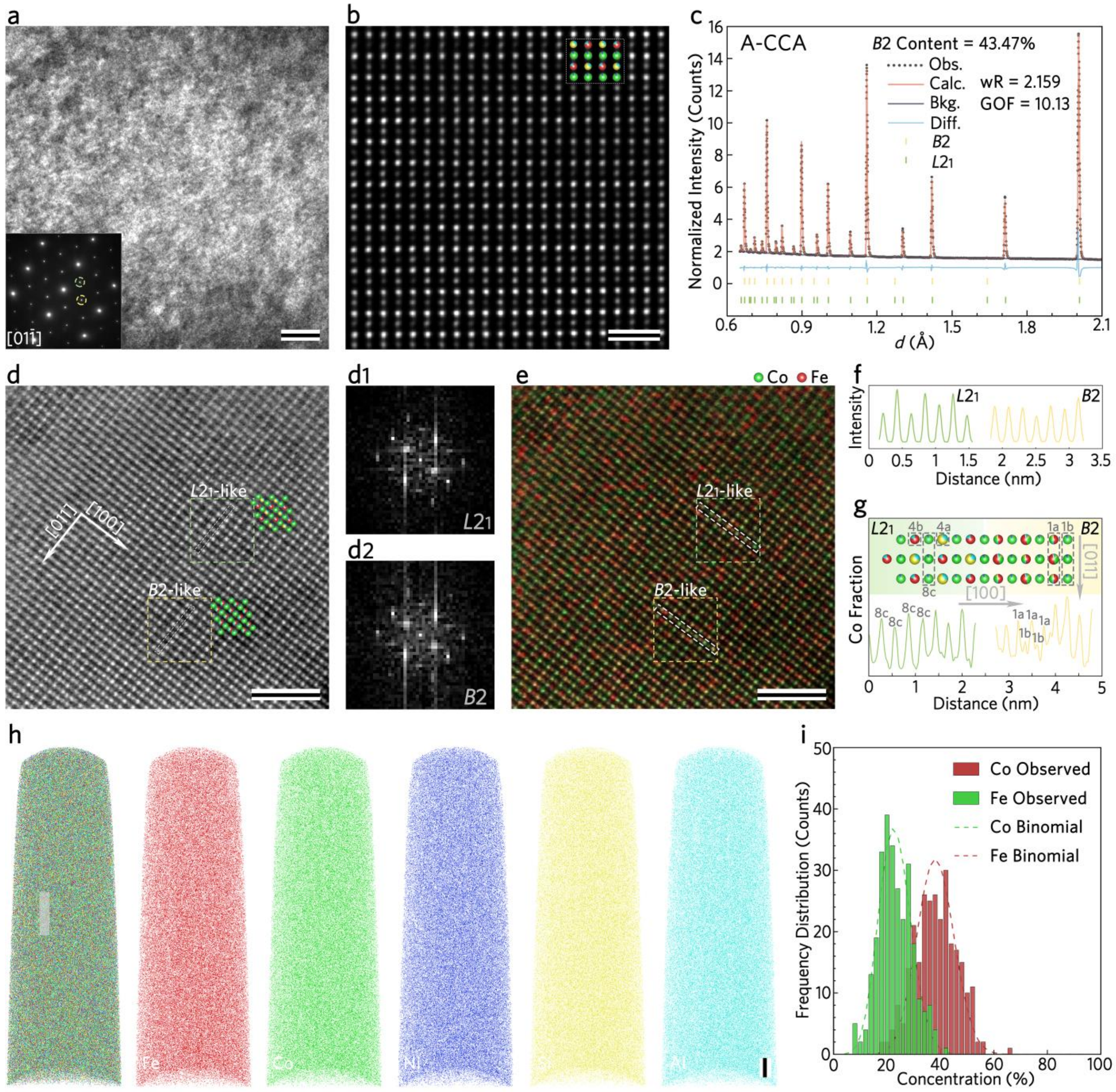

Figure S2. Microstructure of Q-CCA and A-CCA. a) CDF-TEM image of Q-CCA obtained from the (200) superlattice spot (see inset, yellow circle). The entire image appears overexposed, suggesting that it is predominantly composed of ordered superlattices. Scale bar, 10 nm. b) HAADF-STEM image of the $L2_1$ superlattice. The sketch in the upper right illustrates the occupancies. Scale bar, 10 Å. c) Neutron scattering spectrum of A-CCA by Rietveld Refinement method for full pattern fitting. d) HAADF-STEM image of CCO fluctuations showing distinguishable $L2_1$-like and $B2$-like nano-superlattices in real space. Scale bar, 2 nm. d1), d2) Selected area FFT patterns corresponding to $L2_1$-like (d1) and $B2$-like (d2) in (d),

visualizing the discrepancy in the extinction law between $L2_1$ and $B2$ ($B2$ has no (111) superlattice spot). e) The corresponding atomic resolution EDS map in (d), showing the occupancy inclination of different elements (only Fe and Co atoms are shown for readability). Scale bar, 2 nm. f) Z-contrast distribution curves of $L2_1$ and $B2$ along the [011] zone axis in (d), showing the chemical composition difference (see white dashed lines). g) Linear composition scan profiles along the [100] zone axis, obtained from the white dashed lines in (e), showing the rhythmic distribution of different atomic positions (schematic diagram showing the $L2_1$ and $B2$ structures). h) 3D reconstruction map of an APT tip showing no typical chemical element segregation at the submicron scale. Scale bar, 10 nm. i) Frequency distribution of Fe and Co in Figure 1f, showing the phenomenon of Fe/Co segregation at the atomic scale.

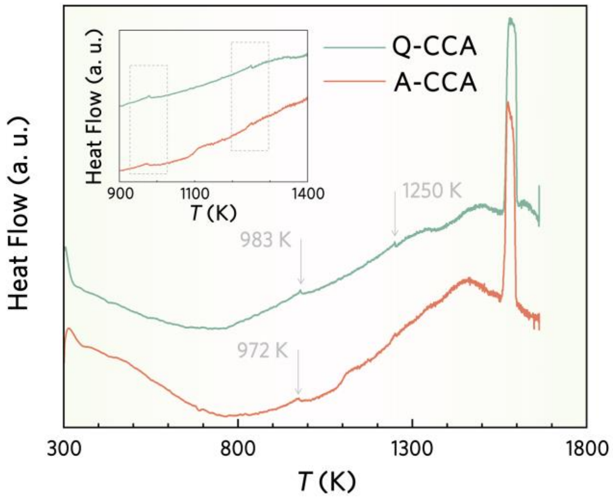

Figure S3. DSC curves of Q-CCA and A-CCA. The inset shows the characteristic of the second order phase transition, indicating the distinct T_c of dual-magnetic-state nature.

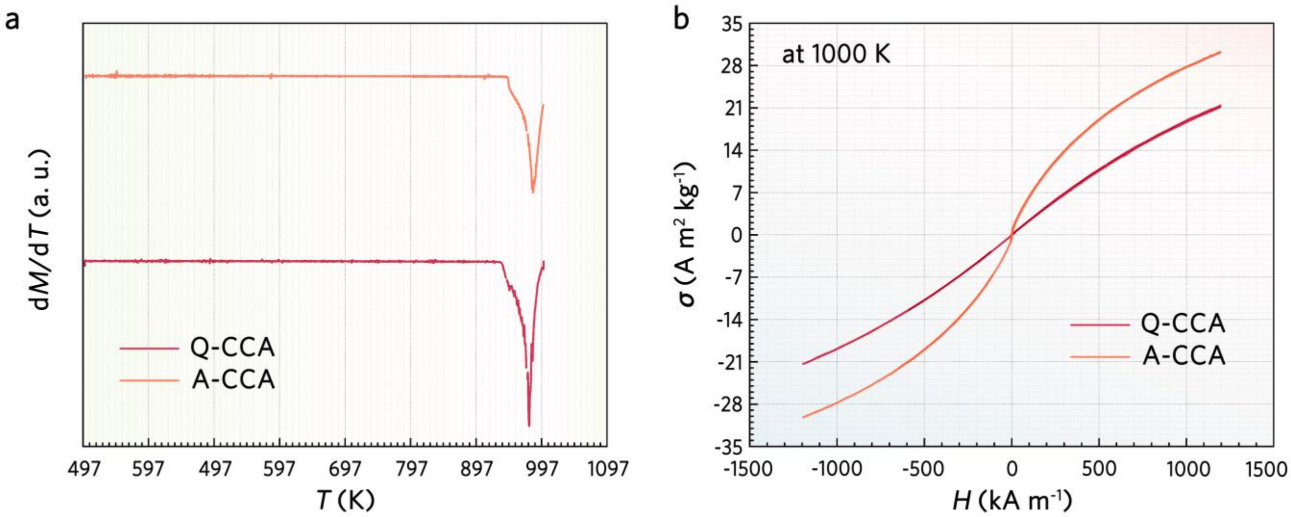

Figure S4. Soft magnetic response of Q-CCA and A-CCA during in-situ heating. a) The d*M*/d*T*-*T* curve, indicating that the T_c of the Co-poor $L2_1$ is about 977 K. The curve is derived by taking the derivative of the *M*-*T* curve shown in Figure 3b, corresponding to a field of 47.76 kA m^{-1}. b) The hysteresis loop at 1000 K. At this point, the ferromagnetism is attributed to the residual Co-rich *B*2.

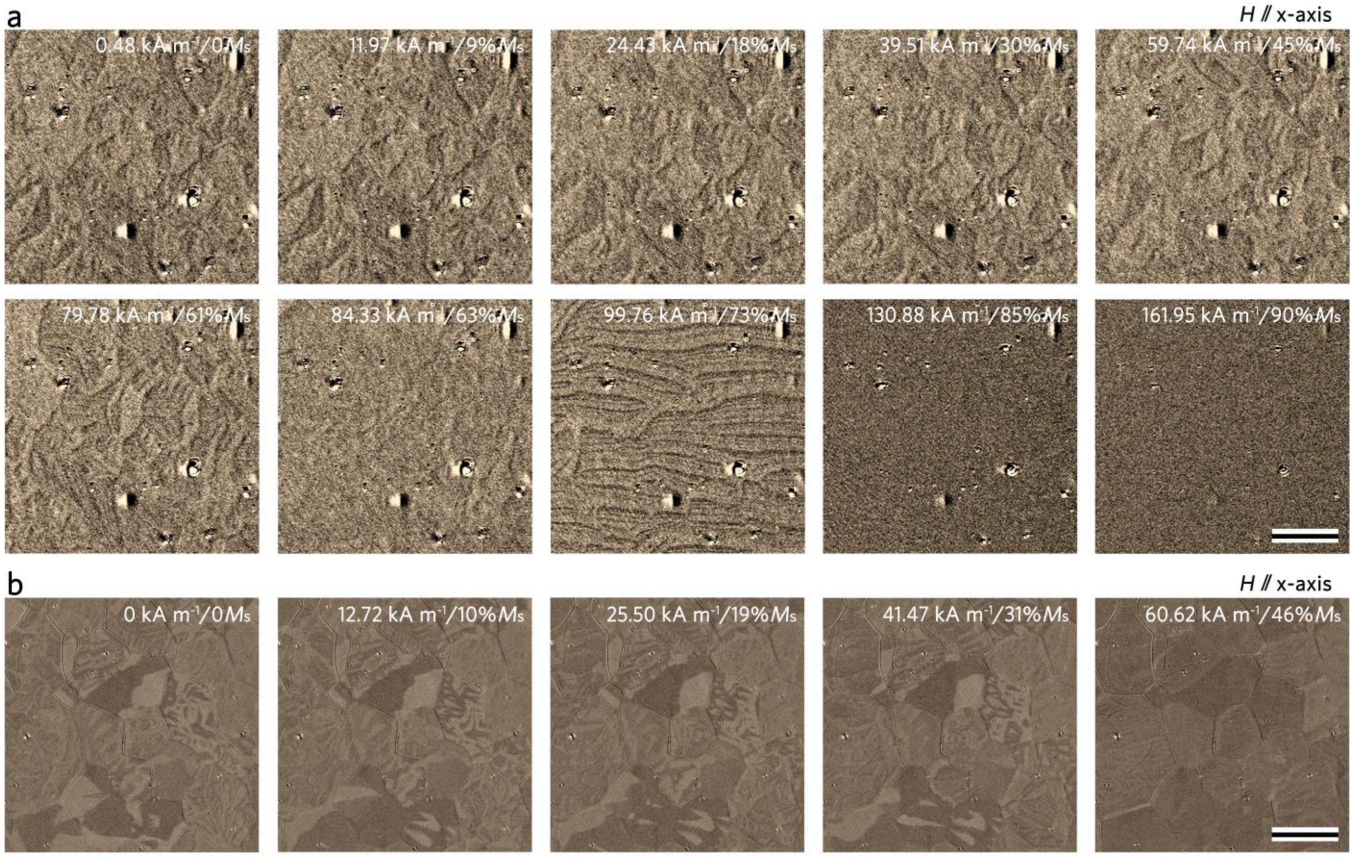

Figure S5. Dynamic evolution of magnetic domains by MOKE. a) Domain changes in Q-CCA. No obvious domain wall movement is observed in the first half of the magnetization process. Note that the striped domain morphology appears at 73%M_s. The direction of field loading is parallel to the plane of observation, specifically to the right. Scale bar, 20 µm. b) Domain changes in C-CCA. Typical domain walls movement can be observed with increasing field. Scale bar, 20 µm.

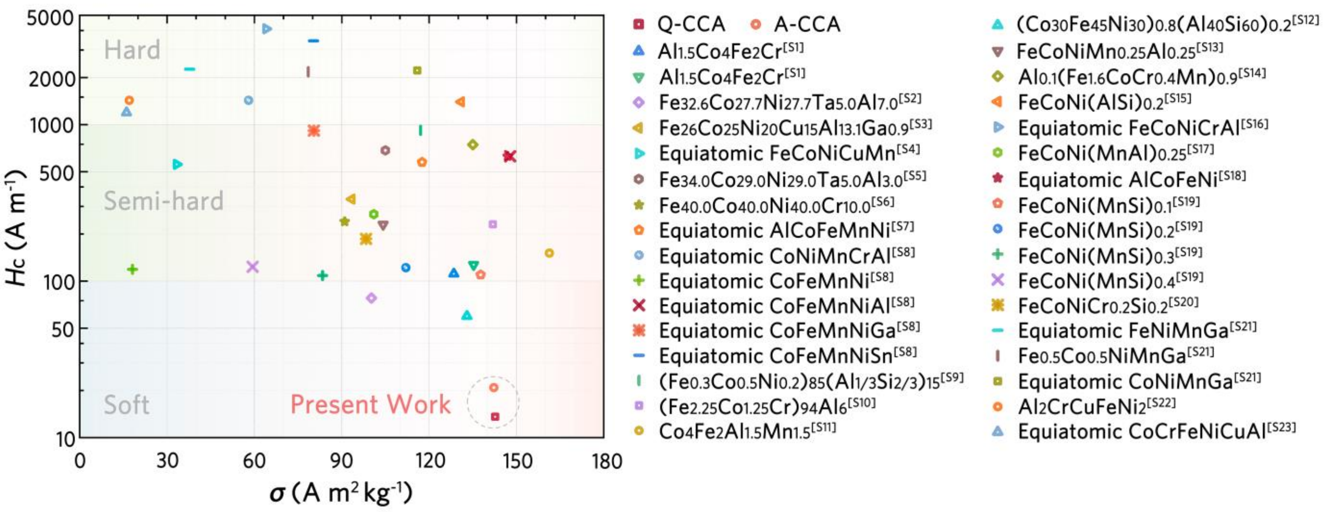

Figure S6. M_s vs. H_c of Q-CCA and A-CCA compared with soft magnetic high-entropy alloys reported in Ref.[S1-S23] It is worth noting that in some of the references it is not labeled whether the ${}_iH_c$ or not, so the vertical coordinate in this Figure is taken as the H_c.

Supporting References

[S1] Y. Ma, Q. Wang, X. Y. Zhou, J. M. Hao, B. Gault, Q. Y. Zhang, C. Dong, T. G. Nieh, *Adv. Mater.* **2021**, *33*, 2006723.

[S2] L. Han, F. Maccari, I. R. Souza Filho, N. J. Peter, Y. Wei, B. Gault, O. Gutfleisch, Z. Li, D. Raabe, *Nature* **2022**, *608*, 310.

[S3] Z. Li, Z. H. Zhang, X. L. Liu, H. X. Li, E. R. Zhang, G. H. Bai, H. Xu, X. G. Liu, X. F. Zhang, *Acta Mater.* **2023**, *254*, 118970.

[S4] M. Harivandi, M. Malekan, S. A. Seyyed Ebrahimi, *Met. Mater. Int.* **2022**, *28*, 556.

[S5] L. Han, Z. Rao, I. R. Souza Filho, F. Maccari, Y. Wei, G. Wu, A. Ahmadian, X. Zhou, O. Gutfleisch, D. Ponge, D. Raabe, Z. Li, *Adv. Mater.* **2021**, *33*, 2102139.

[S6] W. Wang, H. Li, P. Wei, W. Zhang, J. Chen, S. Yuan, Y. Fan, R. Wei, T. Zhang, T. Wang, C. Chen, F. Li, *Mater. Lett.* **2021**, *304*, 130571.

[S7] C. Yang, J. Zhang, M. Li, X. Liu, *Acta Metal Sin-Engl.* **2020**, *33*, 1124.

[S8] T. Zuo, M. C. Gao, L. Ouyang, X. Yang, Y. Cheng, R. Feng, S. Chen, P. K. Liaw, J. A. Hawk, Y. Zhang, *Acta Mater.* ***2017***, *130*, 10.

[S9] Y. Zhang, M. Zhang, D. Li, T. Zuo, K. Zhou, M. Gao, B. Sun, T. Shen, *Metals* **2019**, *9*, 382.

[S10] J. Duan, M. Wang, R. Huang, J. Miao, Y. Lu, T. Wang, T. Li, *Sci. China Mater.* **2022**, *66*, 772.

[S11] W. Gao, Y. Dong, X. Jia, L. Yang, X. Li, S. Wu, R. Zhao, H. Wu, Q. Li, A. He, J. Li, *J. Mater. Sci. Technol.* **2023**, *153*, 22.

[S12] K. X. Zhou, B. R. Sun, G. Y. Liu, X. W. Li, S. W. Xin, P. K. Liaw, T. D. Shen, *Intermetallics* **2020**, *122*, 106801.

[S13] P. Li, A. Wang, C. T. Liu, *J. Alloys Compd.* **2017**, *694*, 55.

[S14] C. Jung, K. Kang, A. Marshal, K. G. Pradeep, J.B. Seol, H. M. Lee, P.P. Choi, *Acta Mater.* **2019**, *171*, 31.

[S15] Y. Zhang, T. Zuo, Y. Cheng, P. K. Liaw, *Sci*. *Rep*. **2013**, *3*, 1455.

[S16] S. G. Ma, and Y. Zhang, *Mater*. *Sci*. *Eng*. *A*. **2012**, *532*, 480.

[S17] P. Li, A. Wang, C. T. Liu, *Intermetallics* **2017**, *87*, 21.

[S18] T. Borkar, V. Chaudhary, B. Gwalani, D. Choudhuri, C. V. Mikler, V. Soni, T. Alam, R. V. Ramanujan, R. Banerjee, *Adv*. *Eng*. *Mater*. **2017**, *19*, 1700048.

[S19] Z. Li, Y. Gu, M. Pan, C. Wang, Z. Wu, X. Hou, X. Tan, H. Xu, *J. Alloys Compd*. **2019**, *792*, 215.

[S20] H. Zhang, Y. Yang, L. Liu, C. Chen, T. Wang, R. Wei, T. Zhang, Y. Dong, F. Li, *J. Magn*. *Magn*. *Mater*. ***2019***, *478,* 116.

[S21] T. Zuo, M. Zhang, P. K. Liaw, Y. Zhang, *Intermetallics* **2018**, *100*, 1.

[S22] T. Borkar, B. Gwalani, D. Choudhuri, C. V. Mikler, C. J. Yannetta, X. Chen, R. V. Ramanujan, M. J. Styles, M. A. Gibson, R. Banerjee, *Acta Mater*. **2016**, *116*, 63.

[S23] K. B. Zhang, Z. Y. Fu, J. Y. Zhang, J. Shi, W. M. Wang, H. Wang, Y. C. Wang, Q. J. Zhang, *J*. *Alloys Compd*. **2010**, *502*, 295.
